# From 2D to 3D: Construction of a 3D Parametric Model for Detection of Dental Roots Shape and Position from a Panoramic Radiograph—A Preliminary Report

**DOI:** 10.1155/2013/964631

**Published:** 2013-03-11

**Authors:** Laura Mazzotta, Mauro Cozzani, Armando Razionale, Sabrina Mutinelli, Attilio Castaldo, Armando Silvestrini-Biavati

**Affiliations:** ^1^Università di Genova, Via Fontevivo 21N, 19125 La Spezia, Italy; ^2^Istituto Giannina Gaslini, Via Fontevivo 21N, 19125 La Spezia, Italy; ^3^University of Pisa, Department of Nuclear Mechanical and Production Engineering, Via Giuntini, Navacchio, 13 56023 Pisa, Italy; ^4^Università di Cagliari, Via Binaghi, 4-09121 Cagliari, Italy; ^5^Trieste University, Piazzale Europa, 1 34128 Trieste, Italy; ^6^Department of Orthodontics, School of Dentistry and Research Center for Material Science and Technology, Università di Genova, Viale Benedetto XV, 6 16132 Genova, Italy

## Abstract

*Objectives*. To build a 3D parametric model to detect shape and volume of dental roots, from a panoramic radiograph (PAN) of the patient. *Materials and Methods*. A PAN and a cone beam computed tomography (CBCT) of a patient were acquired. For each tooth, various parameters were considered (coronal and root lengths and widths): these were measured from the CBCT and from the PAN. Measures were compared to evaluate the accuracy level of PAN measurements. By using a CAD software, parametric models of an incisor and of a molar were constructed employing B-spline curves and free-form surfaces. PAN measures of teeth 2.1 and 3.6 were assigned to the parametric models; the same two teeth were segmented from CBCT. The two models were superimposed to assess the accuracy of the parametric model. *Results*. PAN measures resulted to be accurate and comparable with all other measurements. From model superimposition the maximum error resulted was 1.1 mm on the incisor crown and 2 mm on the molar furcation. *Conclusion*. This study shows that it is possible to build a 3D parametric model starting from 2D information with a clinically valid accuracy level. This can ultimately lead to a crown-root movement simulation.

## 1. Introduction

In the past few years, in orthodontics, the use of invisible appliances like clear aligners has widely increased especially in adult patients. Moreover, other kinds of invisible appliance like *Insignia* [1] (Ormco, West Collins Orange, CA, USA) or *Harmony* (American Orthodontist, Sheboygan, Wisconsin, USA) are now developing: these appliances are not clear aligners but fully customized lingual brackets and wires that can deliver accurate forces [2] to the teeth. However, both clear aligners and invisible lingual brackets set up their treatment plan only on crown information deriving from scanned plaster models. Therefore when a patient is treated with clear aligners, orthodontists do not have either 3D information of dental roots for a treatment plan or root control for an orthodontic movement during therapy [3, 4]. This represents an issue considering that the potential consequence of moving teeth buccally (if roots are in the proximity of the cortical bone) is that these could be moved too buccally, outside the supporting alveolar bone, creating a dehiscence and a potential gingival recession [5]. This is also an issue because roots could be moved one against the other, causing root resorption or root proximity (which is a risk factor for progression of alveolar bone loss [6]). 

It is therefore essential to be able to distinguish position and volume of dental roots in order to prevent root and bone resorptions.

A further problematic aspect which is becoming increasingly significant not only in orthodontics, but also throughout dentistry is radiation protection in terms of the absorbed dose of X-rays in diagnostic imaging. Considering 3D images, cone beam computed tomography (CBCT) has greatly reduced the dose of X-rays absorbed compared to traditional computed tomography (CT), but still (even providing a larger number of information than 2D radiographs), CBCT produces a greater X-ray dose than a panoramic radiograph (PAN) added to a teleradiography [7, 8]. Recently, a study has been published showing the correlation between X-ray diagnostic tests used in dentistry and an increased risk of developing meningioma: this is especially true when X-ray exams are performed repeatedly and in young patients [9].

Panoramic radiographs however have the disadvantages of no constant magnification, image distortion, and narrow image layer. There are in the literature a few quantitative measurement studies, which evaluate tooth length [10] and mesiodistal root angulation [11]: dimensions in the vertical direction may entail a variable amount of magnification that can be as high as 17–27% in the maxillary premolar and 1st molar region with the palatal root having the worst vertical magnification. This is why panoramic radiographs have been of limited use for quantitative studies, preferring 3D imaging or newer technologies [12] instead. Lahreim [13] also measured tooth lengths from panoramic radiographs and showed that measurement error varies from 0.43 to 0.56 mm, indicating that the main source of error was the recognition of reference points.

It is questionable if it is necessary to get the patient to undergo a CBCT or a CT to have 3D information about the shape and volume of dental roots. The aim of this study is to construct a 3D parametric model of teeth, starting from 2D information (the panoramic radiograph of the patient). This has the ultimate goal of simulating a crown-root movement and not only a crown movement during the treatment plan. This has the ultimate goal of simulating a crown-root movement and not only a crown movement during the treatment plan. 

## 2. Materials and Methods

The methodology used in this study to construct and validate the three-dimensional parametric model involved four phases:

 2.1. Data acquisition.
 2.2. Measurements.
 2.3. Construction of the parametric model.
 2.4. Optical integration and teeth segmentation from CBCT.

### 2.1. Data Acquisition

A CBCT and a PAN were acquired from a patient. The patient, a woman, 24 years old and in good health condition, had given consent for the CBCT which was required for the extraction of the lower third molars. She had undergone a previous fixed orthodontic treatment and presented well-aligned arch forms; no periodontal disease was present and neither was any kind of metal appliances (retainers, amalgam restorations, implants) that could interfere with X-rays.

The CBCT was obtained with a Planmeca ProMax 3D unit, (Planmeca Oy, Helsinki, Finland) [14]; CBCT data were stored in DICOM format and processed to generate volumetric representations of anatomical structures. The CBCT scan was necessary for the validation of the study: it was used as a 3D reference model to compare with 2D panoramic measurements. 

The PAN was obtained with a Planmeca ProMax unit (Planmeca Oy, Helsinki Finland); data were stored and processed in DICOM format. The panoramic radiograph was used to choose and measure those parameters which control the parametric model.

### 2.2. Measurements

**Figure 1:**
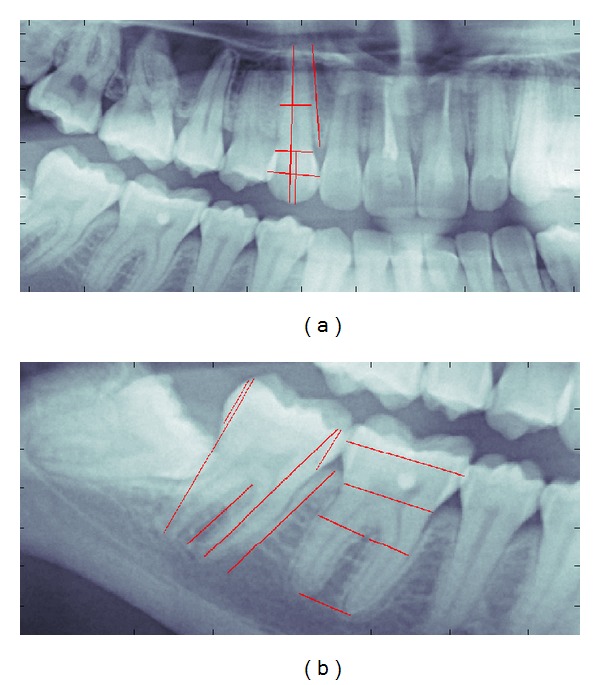
(a) Measures and landmarks on a monoradicular tooth (1.3). (b) Measures and landmarks on multiradicular teeth (4.6, 4.7).

Different parameters were considered for each tooth; these were measured in vivo, from the PAN (Figures 1(a) and 1(b)) and from the CBCT. Measurements were obtained with the already validated method [15] ImageJ 1.46r [16] (Bethesda, Maryland, USA) a digital software for DICOM image processing; in vivo measures were obtained with a digital caliper: no root measures were taken in vivo. The error method was calculated with Dahlberg's equation [17], and it resulted in less than 1 mm for each method. Historically, Dahlberg was the first to provide a formula for repeatability error; the formula originally described is

(1) $$\begin{array}{c} S_{D}=\sqrt{\frac{\sum_{i=1}^{n}d_{i}^{2}}{2n}}, \end{array}$$

where *d* is the difference between the pairs of replicate measurements, *n* is the number of cases, and *S*_*D*_ is the statistical estimate of the “true” error (standard deviation). 

Measures were differentiated according to the number of dental roots (mono- or multiradicular teeth). All the measurements listed below have been carried out by a single person

*Monoradicular*

- Equator (EQU): widest part of the crown; where possible distance between contact points has been considered.
- Coronal height (C-H): highest part of the crown, from gingival azimuth to coronal cusp.
- Cement-enamel junction (CEJ): narrowest part of the crown; in X-ray images a visual transition from cement to enamel was considered.
- Width of the root (RAD): 1 width measurements at half root length.
- Tooth height (T-H): length from the dental apex to the corresponding cusp.

*Multiradicular*

- Equator (EQU): widest part of the crown; where possible, distance between contact points has been considered.
- Mesial coronal height (MC-H): length from the mesial cusp to the CEJ.
- Distal coronal height (DC-H): length from the distal cusp to the CEJ.
- Cement-enamel junction (CEJ): narrowest part of the crown; in X-ray images a visual transition from cement to enamel was considered.
- Distal root width (DRAD): 1 width measurements of the distal root at half root length.
- Mesial root width (MRAD): 1 width measurements of the mesial root at half root length.
- Palatal root width (PRAD): 1 width measurements of the palatal root at half root length.
- Mesial tooth height (MT-H): length from the mesial root apex to the corresponding cusp.
- Distal tooth height (DT-H): length from the distal root apex to the corresponding cusp.
- Palatal tooth height (PT-H): length from the palatal root apex to the corresponding cusp.
- Furcation height (H-FURC): distance between furcation and root apex.

### 2.3. Construction of the Parametric Model

The parametric model was generated with the use of SolidWorks [18] (Dassault Systèmes SolidWorks Corp., Concord, MA, USA), which is a CAD software that can create 2D and 3D surfaces and objects. Several B-Spline curves or “control curves” have been determined: a spline curve is defined by a function composed by a set of polynomials connected to each other. The purpose of these polynomials is to connect a set of points (called nodes of the spline) in a single interval so that the curve is continuous at each point of the range. Spline curves are therefore defined by specific points (nodes) that are determined by the operator according to previously defined parameters. In this study, tooth geometry was modeled through B-spline curves (two-dimensional) and by the aid of NURBS (nonuniform rational basis splines), which are free-form surfaces (three-dimensional). The modeling approach is therefore parametric: measures from the panoramic radiograph have been defined as general control parameters for tooth models. This allows the construction of a 3D virtual model simply by inserting data measured from the panoramic radiograph. In this study we have constructed two models: a model of a monoradicular tooth (maxillary incisor, tooth 2.1) and a model of a multiradicular tooth (mandibular molar, tooth 3.6).

*Monoradicular. *The parametric model of the maxillary incisor was made by 3 closed B-spline curves to define the root width; the crown was modeled with a free-form surface bounded by 4 B-spline curves, which defined its width and its depth. A connection curve was also employed to connect the root with the crown. The control parameters used were

- T-H to define the overall height of the tooth;
- CEJ to define the diameter of the circle connecting the root to the crown;
- EQU to define the width of the crown;
- C-H to set the height of the crown;
- RAD to define root width.

**Figure 2:**
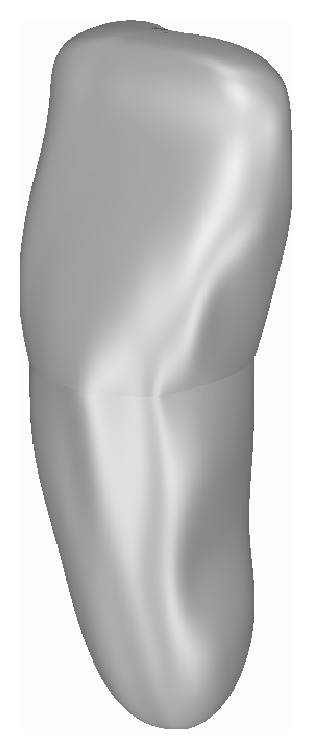
Parametric model of a maxillary incisor.

The relationship between the parameters used and the not specifically defined sizes has been chosen on a statistical basis from the literature (or databases) and maintained as constant as possible (Figure 2)

*Multiradicular*. The mandibular molar parametric model was constructed using revolution surfaces for the roots: these were obtained by using B-Splines as profile curves. The crown was modeled with free-form surfaces bounded by six curves: three closed B-Spline curves for the tooth cross-sections and three open B-Spline curves for the tooth profile, width, and depth. The control parameters used were

- MT-H and DT-H to define the height of the tooth;
- CEJ to define the width of the connection curve between root and crown;
- EQUATOR to define the width of the crown;
- DC-H and MC-H to define the coronal heights;
- DRAD and MRAD to set mesial and distal root widths.

**Figure 3:**
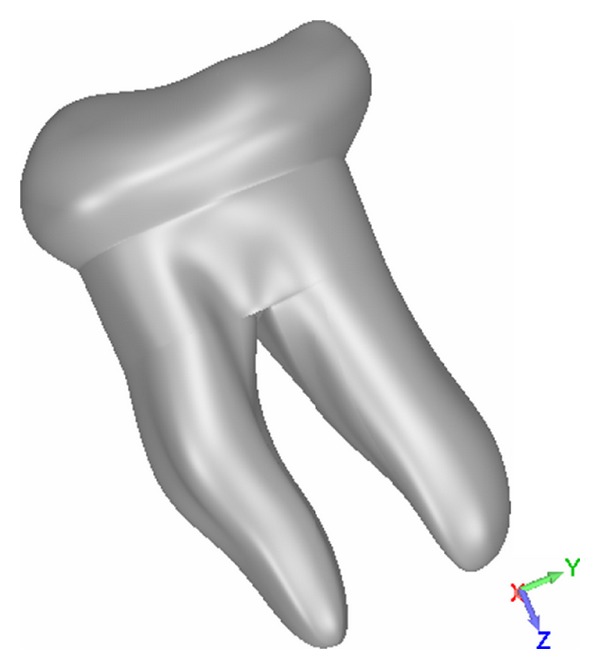
Parametric model of a mandibular molar.

The relationship between the parameters used and the not specifically defined sizes has been chosen on a statistical basis from the literature (or databases) and maintained as constant as possible (Figure 3).

### 2.4. Optical Integration and Teeth Segmentation from CBCT

Segmentation of a digital image is the process of partitioning this image into significant regions. It allows dividing digital images in “sets of pixels”. It is used to obtain more compact representations in order to extract objects or to analyze images. There are different ways to obtain a segmentation, and usually in orthodontics the aim in using segmentation is the partition of dental crowns for the simulation of tooth movement [19, 20].

**Figure 4:**
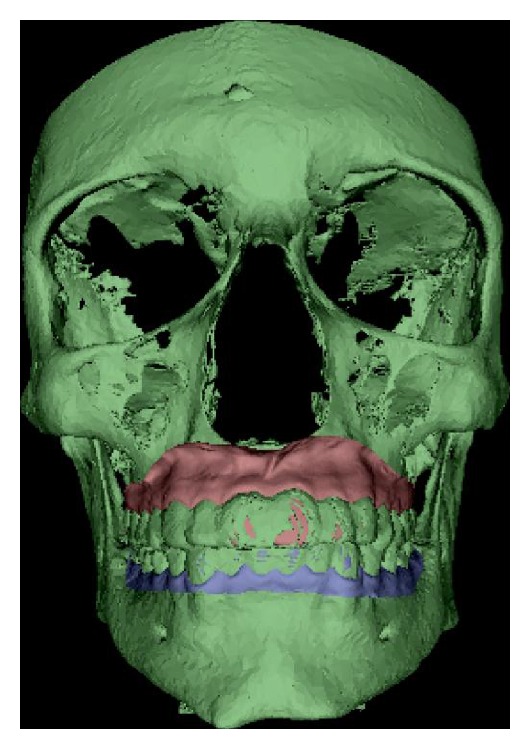
Superimposition of the 3D-scanned models with the CBCT to find the threshold value.

In this study segmentation was obtained through the identification of a “reference threshold value”; the accuracy of 3D models segmented from CBCT is strongly influenced by acquisition parameters and reconstruction settings [21]; a volumetric representation is the result of a threshold value entered by the user according to the visual segmentation of different tissues (it may therefore be subjected to mistakes and inaccuracies). In this study, polyether impressions of the patient were taken, poured in cast, and scanned (using Dental Vision-ScanSystems S.R.L., Cascina, Italia, an optical scanner that has an overall accuracy of 0.01 mm [22–24]): DICOM images have been segmented using 3D scanned models as a reference. The optimal threshold value has been calculated through superimposition of 3D scanned models and CBCT; in such way any difference between the two different scanning technologies has been minimized (Figure 4). 

**Figure 5:**
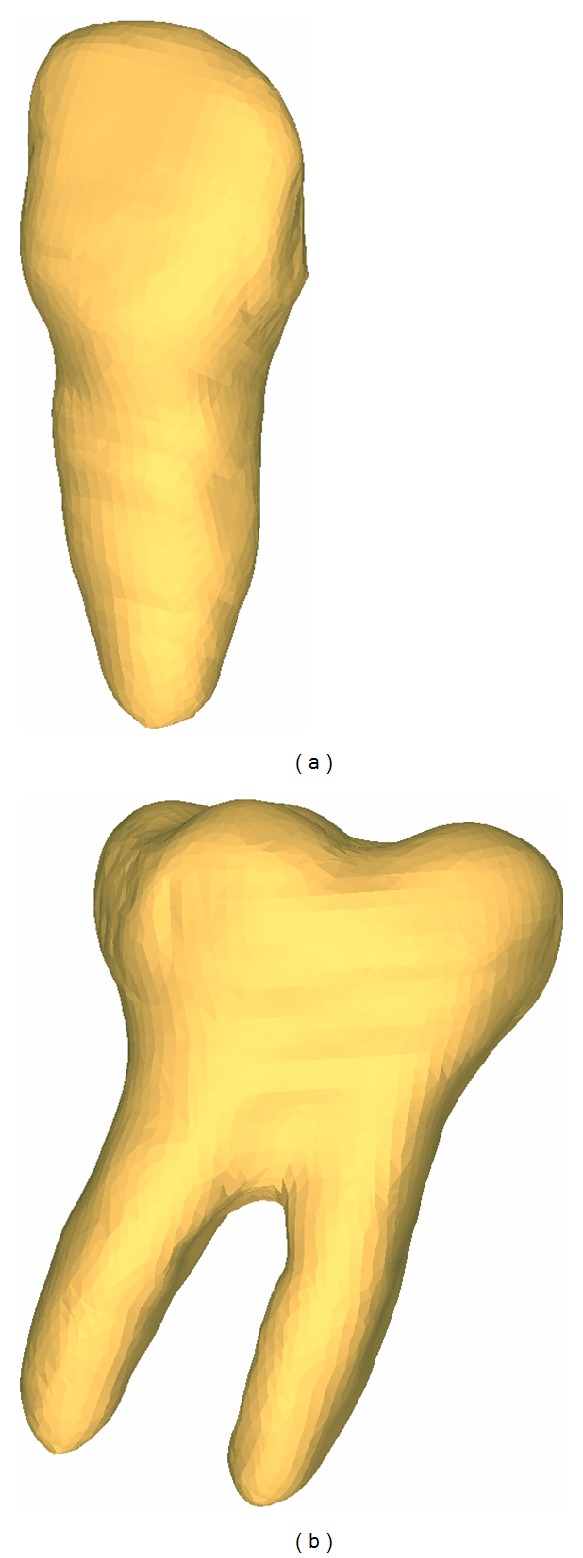
(a) Monoradicular tooth (2.1) segmented and extracted from CBCT. (b) Multiradicular tooth (3.6) segmented and extracted from CBCT.

The purpose of segmentation was to isolate a maxillary incisor and a mandibular molar from the CBCT. The 3D segmented models were superimposed to the parametric models of the corresponding teeth so the accuracy level could be verified. Segmentation was performed using the software Amira 5.4.3 [25] (Visage Imaging Corp, San Diego, CA, USA) (Figures 5(a) and 5(b)), which is a validated method [26, 27].

## 3. Results

### 3.1. Measures

All measurements taken in vivo from the CBCT and from the PAN were analyzed. It was necessary to calculate a scaling factor (0.313854) to make a comparison between CBCT measures and all other measures possible. 

Above all it was necessary to verify values distribution; the Shapiro-Wilk test was applied to CBCT data, to PAN data, and to their difference: it showed that they are not normally distributed; moreover, it was not possible to normalize them through a logarithmic transformation. The Wilcoxon signed-rank test, applied to the outcome of the two variables (CBCT and PAN), was not significant (*P* = 0.5227). The same test was used to verify differences between upper and lower dental arches, and in both cases it is not significant (upper arch *P* = 0.0678, lower arch *P* = 0.3588). The Kruskal-Wallis test, applied to analyze differences between groups of teeth, also was nonsignificant (*P* = 0.0709). CBCT and PAN were found statistically comparable for all root and crown measures, with no difference in maxillary and mandibular arches and in different groups of teeth.

The Shapiro-Wilk test was then applied to in vivo, CBCT, and PAN crown measures: it showed that these data were not normally distributed. Therefore, nonparametric tests were used to compare crown measures. The Kruskal-Wallis test was applied on all four methods, and it was nonsignificant (*P* = 0.1135) meaning that also crown measures are all statistically comparable. 

The Wilcoxon signed-rank test was then applied to crown measures to verify differences in pairs of methods: it still resulted in an insignificance for CBCT and PAN (*P* = 0.9494), for CBCT and in vivo (*P* = 0.0490), and for PAN and in vivo (*P* = 0.0259) measures. 

### 3.2. Model Superimposition and Comparison

**Figure 6:**
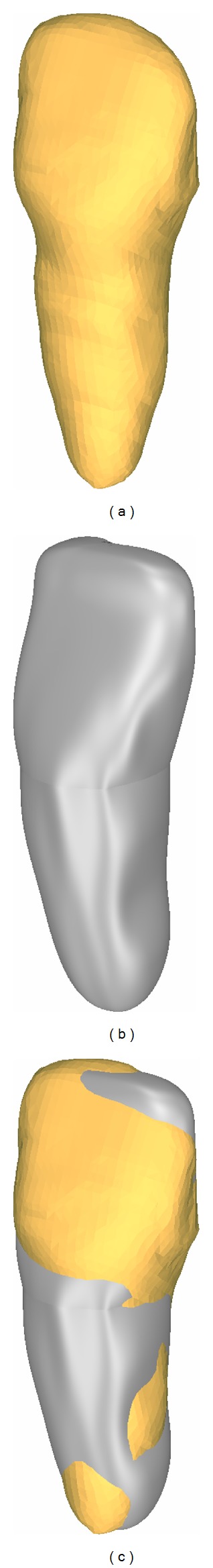
Tooth 2.1. segmented and extracted from cone beam (a), parametric model obtained with PAN measures (b), and superimposition of the models from a buccal vision (c).

**Figure 7:**
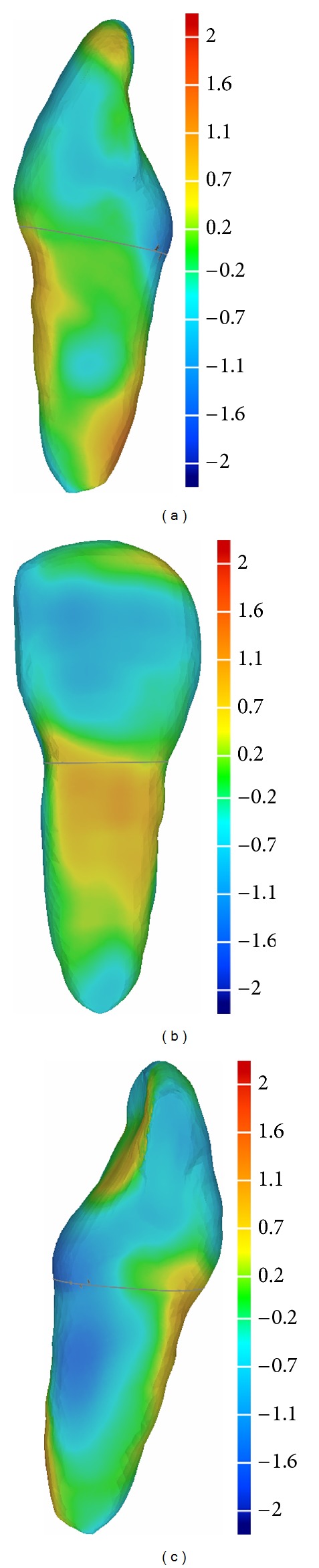
Superimposition and dimensional comparison between the dimensional parametric model and the segmented model of the upper incisor from a mesial view (a), from a buccal view (b), and from a distal view (c).

**Figure 8:**
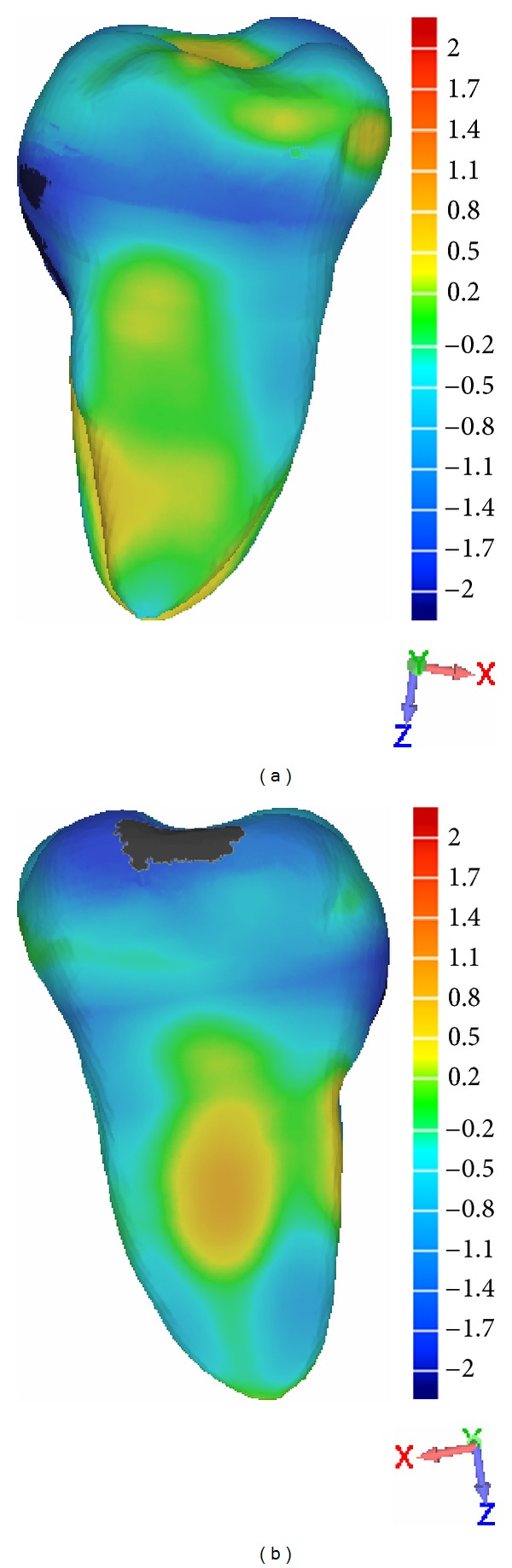
Superimposition and dimensional comparison between the dimensional parametric model and the segmented model of the mandibular molar from a distal view (a), and from a mesial view (b).

Parametric models and segmented models were superimposed using Geomagic [28] (Geomagic Qualify 2012, NC, USA) (Figures 6(a), 6(b), and 6(c)), a dedicated software which enables both the superimposition and the dimensional comparison. During alignment a best-fit algorithm was used to minimize the models overlap. With Geomagic views of the dimensional comparisons were obtained: the figures show the mesial, buccal, and distal views of the maxillary incisor (Figures 7(a), 7(b), and 7(c)) and the mesial and distal views of the mandibular molar (Figures 8(a) and 8(b)).

The dimensional comparison must be interpreted according to colors: in presence of warm colors there is an area of excess parametric model with respect to the segmented model; cold colors, on the other hand, represent a lacking area of the parametric model compared to the segmented model.

The maxillary incisor model on the mesial side (Figure 7(a)) has an overall gap of 0.8 mm compared to the segmented model; the discrepancy increases at the apex level with a model excess, which arrives up to 1.1 mm. On the buccal side (Figure 7(b)), there is an overall discrepancy of about 0.8 mm. The distal side presents a discrepancy that goes from 0 to 1.6 mm: maximum error is at the cement-enamel junction level.

The mandibular molar model (Figures 8(a) and 8(b)) show an overall root discrepancy of 0.7 mm. At crown level it presents areas with a 0–0.5 mm gap (lingual side) and areas where the discrepancy arrives up to 2 mm (vestibular and occlusal side).

## 4. Discussion

To define the accuracy level it is necessary to analyze errors, which may be intrinsic to the method: data acquisition errors, segmentation process errors, and errors due to model alignment.

Regarding the data acquisition process, according to the literature, CBCT can be used for dental measurements with an acceptable accuracy level [29], while images from a panoramic radiograph may be distorted [30]. In the present study reference points and measuring methods proved to be reliable: Dhalberg's equation resulted in less than 1 mm for each method. Moreover, the risk of panoramic radiograph deformation had been taken into account: all measurements were compared with CBCT and statistics resulted nonsignificant, as a consequence the parametric model could be constructed.

Parametric models of only two teeth were constructed (this is a preliminary study) but all teeth were measured: this was necessary to provide a sufficient amount of data for a valid statistic-test on panoramic radiograph accuracy. 

Segmentation was performed with particular attention to the threshold value: the most accurate threshold value possible was obtained by superimposing the scanned model and the CBCT. Furthermore, the literature shows that the difference between the volume of extracted teeth and CBCT-segmented teeth varies from −4% to 7% [31], a value that is clinically acceptable if the purpose is the simulation of tooth movement.

Finally, the superimposition and the dimensional comparison of the models were performed using a best-fit algorithm, which minimizes models alignment error. The models alignment error is therefore the minimum possible and depends just on how different the models are between them: if the models were the same, the error would be zero. The alignment error is comparable to the error detected by the comparison of the two models superimposed (in this case, from 0 to 1.6 mm for the incisor and from 0 to 2 for the molar).

In this study we obtained only mesio/distal measurements: we are not able to achieve buccal/lingual measures from a pan. The only way to verify the buccal/lingual accuracy level is with the superimposition of the two models (Figures 7(a), 7(b), 7(c), 8(a), and 8(b)) that show that the model can be superimposed to a segmented tooth with a clinically valid accuracy at root level. The crucial point of this study was the root model: the crown model was not developed as much in detail because it will be integrated with a more precise 3D scanned model.

Recently Kihara et al. [32] have published a study the aim of which is the reconstruction of dental roots directly on the scanned model: their study presents more accurate and reliable results than those presented in this study (average discrepancy of 0.025 ± 0.007 mm), but their method contemplates the use of CBCT in every patient and therefore an increased dose of radiation that should be justified [8]. 

Recent studies have also shown that a CBCT needs to be interpreted by a person trained in advanced interpretation techniques in radiology because of the wide scope of incidental findings noted on these scans [33]: this methodology would be an easier and safer way both for clinicians and patients to access 3D root information.

The parametric model shown in this study was comparable with the segmented model with a final, clinically acceptable error; however, it is simplified: any curves or root malformations are undetectable. There are still many aspects that should and will be investigated. The patient had well-aligned teeth; how would the parametric model work if there were rotations in teeth or overlaps due to crowding or ectopic eruption? What about patients that do not have a perfectly positioned panoramic image capture? And what about measures for those teeth that according to the literature have the worst vertical magnification in panoramic radiographs? We do believe that having the scanned plaster models and merging them with the parametric models will help reduce root errors even in displaced teeth. More studies are necessary and are already underway to make the construction of more accurate and reliable models possible, in order to construct and validate all teeth from all quadrants. 

## 5. Conclusion

This preliminary study showed a new method, which makes possible the construction of a three-dimensional parametric model. Above all, the importance of this method is the reduction of the radiation dose because it provides 3D information of dental roots without using CBCT, with benefits for patients' health. The models have an accuracy level that is statistically and clinically acceptable; however, the quality of the crown of the parametric model is poor, and room for improvement still exists. Further studies are necessary and are underway in order to build models for every tooth and to reconstruct the entire dental arch.
